# CRP gene variation affects early development of Alzheimer's disease-related plaques

**DOI:** 10.1186/1742-2094-8-96

**Published:** 2011-08-11

**Authors:** Eloise Helena Kok, Mervi Alanne-Kinnunen, Karita Isotalo, Teemu Luoto, Satu Haikonen, Sirkka Goebeler, Markus Perola, Mikko A Hurme, Hannu Haapasalo, Pekka J Karhunen

**Affiliations:** 1 School of Medicine, University of Tampere and Centre for Laboratory Medicine, Tampere University Hospital, Tampere Finland; 2 Wihuri Research Institute, Helsinki, Finland; 3 Department of Neurosciences and Rehabilitation, Tampere University Hospital, Tampere, Finland; 4 National Institute for Health and Welfare, Tampere, Finland; 5 Department of Chronic Disease Prevention, National Institute for Health and Welfare, Unit of Public Health Genomics, Helsinki, Finland; Institute for Molecular Medicine Finland FIMM, University of Helsinki, Helsinki, Finland; Department of Medical Genetics, University of Helsinki, Helsinki, Finland

## Abstract

**Introduction:**

We used the Tampere Autopsy Study (TASTY) series (n = 603, age 0-97 yrs), representing an unselected population outside institutions, to investigate the pathogenic involvement of inflammation in Alzheimer's disease-related lesions.

**Methods:**

We studied senile plaque (SP), neurofibrillary tangles (NFT) and SP phenotype associations with 6 reported haplotype tagging single nucleotide polymorphisms (SNPs) in the CRP gene. CRP and Aβ immunohistochemistry was assessed using brain tissue microarrays.

**Results:**

In multivariate analyses (age- and APOE-adjusted), non-neuritic SP were associated with the high-CRP TA-genotype (3.0% prevalence) of rs3091244 and CA-genotype (10.8%) of rs3093075 compared to common genotypes. Conversely, the low-CRP C allele (39.3%) of rs2794521 reduced the risk of harbouring early non-neuritic SP, compared to the TT genotype. CRP haplotype TAGCC (high) associated with non-neuritic SP, whereas haplotype CCGCC offered protection. TT genotypes (high) of rs3091244 and rs1130864 were associated with CRP staining. There were no associations between SNPs or haplotypes and NFT. CRP staining of the hippocampal CA1/2 region correlated with Aβ staining.

**Conclusions:**

CRP gene variation affects early SP development in prodromal Alzheimer's disease, independent of APOE genotype.

## Background

The only method for definitive diagnosis of Alzheimer's disease (AD) to date is postmortem examination of the brain. Current understanding indicates that the neuropathological hallmarks, senile plaques (SP) and neurofibrillary tangles (NFT) develop within the brain, interrupting neuronal signalling and causing the irreversible symptoms of memory impairment and gradual cognitive decline [1,2]. Efforts to prevent or slow the disease are hampered by a lack of understanding as to how these neuropathological hallmarks develop and actually cause the disease - if they do.

There are two forms of AD - familial and sporadic - of which the sporadic is much more common, comprising 96% of all cases. Familial AD (FAD) is mostly caused by mutations in 3 particular genes (amyloid precursor protein, presenilin 1 and presenilin 2) [3], which are directly related to the formation of SP. This has lead researchers to believe that SP are the main culprit in all forms of AD. Many studies have revealed environmental and genetic factors that affect the risk of sporadic AD, such as exercise, education level and the ε4 allele of *APOE *[4].

At present, the apolipoprotein E (*APOE*) ε4 allele is the only commonly accepted gene known to confer increased risk for sporadic AD, whilst the rare ε2 allele is believed to convey protection. Various studies have found ORs of between 2 and 8, as well as lowering the age of onset, with ε4 allele dosage [5,6]. Recently, genome wide association studies [7-9] have revealed some lower impact genes that may increase AD risk, possibly accounting for a part of the remaining unexplained ~50% of genetic risk effects. Many other genes have also been suggested to increase the risk of AD, but the evidence has been conflicting, with *APOE *being the only consistent association.

The possible connection between AD and inflammation was ignited by a study [10] showing a reduced incidence of AD in a cohort of rheumatoid arthritic patients taking non-steroidal anti-inflammatory drugs (NSAIDs), however other studies have disputed this connection [11]. New research [12-14] supports this, as many inflammatory markers have been found localised with the neuropathological characteristics of AD; these include neuroinflammatory cells, astrocytes, and microglia. Recent genome wide association studies have also shed light on this, with inflammatory genes being put in the spotlight [9]. It has also been suggested that chronic inflammation in the brain from various bacterial/viral diseases could contribute to the disease [15,16]. Interactions between inflammatory gene polymorphisms and invading pathogens have also been proposed to participate in disease manifestation [17]. The question remains, however, whether the inflammatory processes are a cause or consequence of the disease, as a majority of previous studies have been conducted in advanced stage AD cases.

C-reactive protein (CRP) is an acute phase inflammatory marker found in plasma, primarily produced by the liver to combat pathogens through activation of immune responses [18]. Additionally, CRP activates the cleanup of cellular debris through its action as a pattern recognition receptor involving calcium-dependent ligand binding [19]. Its role in AD has already been suggested by work by Yasojima et al., which showed that CRP production is upregulated in affected areas of AD brains [20].

Some single nucleotide polymorphisms (SNPs) of the *CRP *gene have been shown to associate with higher CRP levels. One of the most influential of these polymorphisms, identified in a genome-wide association study, was rs3091244 (T and A alleles), as well as others; rs1130864 (T allele), rs1205 (G allele) and rs3093075 (C allele)[21-23]. The SNP rs2794521 (T allele) has been reported to increase transcription of the *CRP *allele [24,25]. Haplotypes associated with 2-3-fold increases in CRP levels correlate with poorer survival in general of elderly subjects [22]. Lower CRP levels have been associated with the C allele of SNP rs1800947 [21,26,24,27] and common haplotypes of the gene are also associated with serum CRP concentration [24].

We have shown previously that accumulation of AD neuropathological lesions is unexpectedly common, with 31.1% of individuals living outside institutions having SP and 42.1% having NFT [28]. This accumulation starts already around 30 years of age, especially among the carriers of the *APOE *ε4 allele, reaching an occurrence of almost 100% in the oldest. Other studies have also shown associations with the *APOE *ε4 allele and both SP and NFT [29,30].

We hypothesised that individuals with *CRP *genotypes associated with higher CRP production would be more likely to show development of SP already in the prodromal phase before the development of clinical AD. At the least, these phenomena might participate in the early stages in the development of the lesions. We explored potential associations between the *CRP *gene and the brain changes commonly linked to AD in a large autopsy cohort representing a population living outside institutions, of which the majority were non-AD patients who died mainly out-of-hospital. As far as we are aware, this is the first study that has looked at the association between AD pathology and CRP, both at a genetic and cellular level.

## Methods

### Cohort

The Tampere Autopsy Study (TASTY) cohort comprises 603 men and women aged 0 - 97 years who were subjected to medico-legal autopsy and generally died out-of-hospital in Finland during the years 2002-2004, representing around 4% of deaths in the Tampere region. None died of AD causes, although 6 (< 1%) were clinically diagnosed with AD during life, 22 (3.7%) were demented and 10 (1.7%) had memory problems. Recorded causes of death are given in table 1; more detailed causes of death are not available. Further data on illnesses and/or medication use during life are not accessible to the researchers. Autopsies were performed by the department of Forensic Medicine at the University of Tampere and data pertaining to the cases were obtained from doctors and family members where possible. The study was approved by the Board of Medicolegal Affairs of Finland.

**Table 1:** The Tampere Autopsy Study (TASTY) characteristics

*Number of cases*	603
*Gender*	
*Males*	388 (64.3%)
*Females*	215 (35.7%)
*Age (years)^1^*	62.7 (range 0 - 96.7)
*Cause of Death*	
*Disease*	340 (56.5%)
*Accident*	177 (29.5%)
*Suicide*	72 (12.0%)
*Homicide*	3 (0.5%)
*Unknown*	9 (1.5%)
*Brain Mass (g)^1^*	1408.1 (range 427 - 1910)
*Dementia Status*	
*Normal*	570 (94.5%)
*AD*	6 (0.9%)
*Dementia*	16 (2.7%)
*Memory Problems*	10 (1.7%)
*Parkinson's Dis*	1 (0.2%)
*APOE Genotype*	
*APOEε3ε3*	356 (59.2%)
*APOEε2ε3, ε2ε2*	58 (9.7%)
*APOEε4+*	187 (31.1%)
*SP Presence*	
*No*	381 (68.9%)
*Yes*	172 (31.1%)
*CERAD score*	
*< 0%*	379
*0 - 1.053%*	85
*1.053% +*	85
*NFT Presence*	
*No*	280 (57.9%)
*Yes*	204 (42.1%)

*^1 ^*- statistical mean.

### Senile plaques and neurofibrillary tangles

SP and NFT assessments were made as previously described [28]. A large number (70%) of cases had 'no SP' and using this skewed data as a continuous variable would make analyses invalid; therefore we categorised the SP into the following categorisations: ≥1 SP (yes/no), and SP typing (no SP, non-neuritic SP (diffuse/primitive), neuritic SP (classic/burnt out)). Analyses also investigated SP density in a semi-quantitative manner, dividing SP counts into 'no SP', 'sparse SP', 'moderate SP' and 'frequent SP', comprising a scoring system based on the CERAD protocol (but without age adjustment). We categorised NFT as: ≥1 NFT (yes/no). NFT and SP were defined by a neuropathologist assessing grid regions of complete brain samples on Bielschowsky-stained slides of frontal cortex (SP) and hippocampus (NFT) in each case. In our cohort, females were older on average by 10 years, causing the category of gender to represent age, however analyses showed similar results when split by gender. Therefore gender was excluded as a covariate in our analyses.

### Tissue microarrays

Tissue microarrays (TMAs) were also constructed (as described in [28]), to allow easier and simultaneous analysis of multiple cases, and held approximately 10-14 cases per block. TMAs were utilised for immunohistochemistry for CRP and Aβ staining. Brain regions that were incorporated into the TMAs were the hippocampal regions CA1, CA2, CA3, and CA4; cerebellum, neocortex (frontal lobe), gyrus cinguli and cerebrum (white matter). Technical difficulties and sample damage precluded inclusion of all TASTY cases, but 92.5% were incorporated.

### Genotyping

CRP genotyping was performed at Biomedicum, Helsinki (MA) on the Sequenom MassArray system with the homogeneous Mass Extension (hME) reaction (Sequenom, San Diego, USA) for 6 reported haplotype tagging single nucleotide polymorphisms (SNPs), including rs2794521 (T > C), rs3091244 (C > T > A), rs1800947 (G > C), rs1130864 (C > T), rs1205 (C > T) and rs3093075 (C > A). Haplotyping was calculated with 5 SNPs (SNP order: rs2794521, rs3091244, rs1800947, rs1130864 and rs1205; rs3093075 was excluded as it produced too many low prevalence haplotypes) using the PHASE program [31,32] (version 2.1.1) and indicated five haplotypes with prevalence above 5%.

### Immunohistochemistry

Fluorescent immunohistochemical (F-IHC) staining was performed on the TASTY-TMAs in the hippocampal CA1/2 area and utilised DAPI (Sigma-Aldrich, Germany), rabbit anti-CRP (BioLegend, USA), mouse anti-Aβ (Acris Antibodies, Germany), anti-mouse IgG FITC conjugated (Novus Biologicals, USA), anti-rabbit IgG rhodamine conjugated (Antibodies-online, Germany), all according to manufacturer's instructions. For analyses, cases were assessed as positive or negative for staining.

### Statistics

Statistical analyses were performed with an SPSS program (version 14.0). Analyses for CRP SNPs and haplotypes used the most common genotype or previously reported 'risk' allele as the reference group and included APOE4 carriership and age as covariates where possible. Their associations were analysed using logistic regression. Chi square analysis was used to determine association with IHC staining. False discovery rate (FDR) multiple correction calculations were performed assuming there were 11 independent tests (6 SNPs and 5 haplotypes), using the calculation below and assuming an FDR value of < 0.05 was acceptable.

## Results

### Cohort

The Tampere Autopsy Study (TASTY) (Table 1) consisted of 603 autopsy cases (35.7% females) of subjects who died mainly out-of-hospital over a three year period. Data on memory problems or possible dementia were collected from hospital records and/or next of kin. Of the series 558 cases (92.5%) were included in the brain tissue microarray (TMA) construction. Not all samples were included due to data discrepancies, technical issues and sample decay/damage.

### Senile plaques and neurofibrillary tangles

Senile plaque (SP) frequency was available for 553 (90.9%), and neurofibrillary tangle (NFT) counts for 484 (80.3%). Both lesions were positively associated with age [28].

### Genotyping

*APOE *genotyping was performed on 601 cases and *CRP *genotypes were acquired for 537 cases (89%). *APOE *and *CRP *genotyping indicated that there were no significant differences in the distribution of allele frequencies in each age group, and that they followed Hardy-Weinberg proportions.

### Associations between genotypes and neuropathological lesions

Univariate logistic regression analysis showed that the SNP rs2794521 (p = 0.067) was associated with SP prevalence (yes/no SP presence). However, including age and *APOE4 *carriership as covariates weakened the association (p = 0.096).

When we took into account the phenotype of SP (Table 2), two high-CRP level-linked SNPs - rs3091244 (TA carriers; OR 6.7, p = 0.007) and rs3093075 (CA carriers; OR 3.5, p = 0.003) - appeared to convey increased risk for early non-neuritic SP compared to no SP. There was also a tendency towards increased risk for late neuritic SP (OR 4.5, p = 0.072; OR 2.1, p = 0.080, respectively).

**Table 2:** Multivariate logistic regression for SP type (no SP - reference group, non-neuritic SP and neuritic SP) and association with *CRP *SNPs (*APOE4 *carriership and age were included as covariates)

					Non-Neuritic SP					Neuritic SP				
		**Assoc**.	**Total**	**Prev %**	**Affected (%)**		**OR**	**CI**	***p***	**Affected (%)**		**OR**	**CI**	***p***

rs2794521	TT*	T allele - high	321	60.8	36	11.2	1	Ref	-	68	21.2	1	Ref	-
	CC		25	4.7	2	8.0	0.673	0.142 - 3.200	0.619	8	32.0	1.265	0.410 - 2.272	0.683
	CT		182	34.5	13	7.1	0.433	0.197 - 0.952	0.037^a^	26	14.3	0.600	0.317 - 1.138	0.118
														
rs3091244	CC*	T & A alleles - high	179	33.7	18	10.1	1	Ref	-	32	17.9	1	Ref	-
	TT		73	13.7	2	2.7	0.290	0.063 - 1.334	0.112	19	26.0	1.829	0.786 - 4.254	0.161
	TA		16	3.0	5	31.3	6.717	1.673 - 26.978	0.007^a^	3	18.8	4.535	0.873 - 23.555	0.072
	CA		41	7.7	7	17.1	1.771	0.606 - 5.172	0.296	9	22.0	2.117	0.730 - 6.139	0.167
	AA		3	0.6	0	0	.	.	.	0	0	.	.	0.998
	TC		219	41.2	20	9.1	0.819	0.384 - 1.744	0.604	40	18.3	1.179	0.589 - 2.361	0.642
														
rs1800947	GG*	C allele - low	457	86.4	43	9.4	1	Ref	-	89	19.5	1	Ref	-
	CC		5	0.9	1	20.0	7.107	0.419 - 120.535	0.175	2	40.0	3.814	0.160 - 90.798	0.408
	GC		67	12.7	7	10.4	1.428	0.579 - 3.526	0.439	12	17.9	0.700	0.270 - 1.813	0.463
														
rs1130864	CC*	T allele - high	220	42.2	25	11.4	1	Ref	-	40	18.2	1	Ref	-
	TT		72	13.8	2	2.8	0.258	0.058 - 1.154	0.076	19	26.4	1.645	0.738 - 3.666	0.224
	TC		229	44.0	24	10.5	0.898	0.461 - 1.748	0.751	43	18.8	1.185	0.630 - 2.229	0.599
														
rs1205	TT*	C allele - high	65	12.3	9	13.8	1	Ref	-	12	18.5	1	Ref	-
	CC		224	42.5	15	6.7	0.397	0.154 - 1.025	0.056	51	22.8	1.492	0.584 - 3.814	0.403
	CT		238	45.2	28	11.8	0.675	0.281 - 1.623	0.380	40	16.8	0.949	0.363 - 2.478	0.914
														
rs3093075	CC*	C allele - high	469	88.7	39	8.3	1	Ref	-	91	19.4	1	Ref	-
	AA		3	0.6	0	0	.	.	.	0	0	.	.	.
	CA		57	10.8	12	21.1	3.492	1.545 - 7.894	0.003^a^	12	21.1	2.143	0.914 - 5.022	0.080

* denotes the most common homozygous genotype acting as the reference group in analyses.

. denotes the values were unable to be computed.

^a^denotes statistically significant values.

Non-neuritic SP are diffuse and primitive SP grouped together, neuritic SP are classic and burnt out SP grouped together; as measured by a neuropathologist.

Prev % refers to prevalence of alleles.

Assoc. refers to associations with CRP levels.

CRP = c-reactive protein gene, SNPs = single nucleotide polymorphisms, SP = senile plaques, OR = odds ratio, CI = confidence interval, *p *= p value.

On the contrary, carriers of the low-CRP level-linked C allele of SNP rs2794521 (OR 0.46, CI 0.22 - 0.96, p = 0.039) were less likely to have non-neuritic SP, derived from an association with the common CT genotype (OR 0.43, p = 0.037). A trend towards the same associations was seen with neuritic SP. Conversely, the high-CRP level SNPs rs1130864 (TT carriers; OR 0.26, p = 0.076) and rs1205 (CC carriers; OR 0.39, p = 0.056) showed a non-significant trend towards protection for non-neuritic compared to no SP.

In multivariate logistic regression, *CRP *haplotypes composed of alleles related to high-CRP levels, such as TAGCC, were associated with presence of non-neuritic SP (OR 2.99, p = 0.007), significantly increasing the risk of occurrence (Table 3). On the contrary, haplotype carriership of alleles linked with lower CRP levels, such as CCGCC, reduced (OR 0.45, p = 0.034) the likelihood of possessing non-neuritic SP. Similar, but-non significant tendencies towards these associations were also seen for both haplotypes and neuritic SP.

**Table 3:** Multivariate logistic regression results for SP type (no SP - reference group, non-neuritic SP and neuritic SP) and association with *CRP *haplotypes (*APOE4 *carriership and age were included as covariates)

					Non-Neuritic SP					Neuritic SP				
		**Assoc**.	**Total**	**Prev %**	**Affected (%)**		**OR**	**CI**	***p***	**Affected (%)**		**OR**	**CI**	***p***

TTGTC	Yes*	High-CRP	306	37.0	26	8.5	1	Ref	-	62	20.3	1	Ref	-
(1)	No		225		26	11.6	1.402	0.740 - 2.656	0.300	41	18.2	0.776	0.435 - 1.383	0.390
TCGCC	No*	No assoc.	516		52	10.1	1	Ref	-	96	18.6	1	Ref	-
(3)	Yes		15	1.2	0	0.0	.	.	.	7	46.7	4.124	0.700 - 24.278	0.117
TCGCT	No*	No assoc.	282		22	7.8	1	Ref	-	61	21.6	1	Ref	-
(4)	Yes		249	30.0	30	12.0	1.397	0.736 - 2.651	0.307	42	16.9	0.686	0.386 - 1.217	0.197
TCCCT	No*	Low-CRP in females	459		44	9.6	1	Ref	-	89	19.4	1	Ref	-
(5)	Yes		72	6.6	8	11.1	1.545	0.655 - 3.644	0.321	14	19.4	0.775	0.312 - 1.923	0.582
TAGCC	No*	High-CRP	471		40	8.5	1	Ref	-	91	19.3	1	Ref	-
(6)	Yes		60	5.2	12	20.0	2.985	1.342 - 6.638	0.007^a^	12	20.0	1.809	0.785 - 4.167	0.164
CCGCC	No*	Low-CRP in males	324		37	11.4	1	Ref	-	69	21.3	1	Ref	-
(7)	Yes		207	19.5	15	7.2	0.453	0.218 - 0.941	0.034^a^	34	16.4	0.680	0.376 - 1.228	0.201

* denotes the most common haplotype acting as the reference group in analyses.

. denotes the values were unable to be computed.

^a^denotes statistically significant values.

Numbers in brackets referring to our own number allocation system for haplotypes.

Haplotypes consist of SNPs rs2794521 (T > C), rs3091244 (C > T > A), rs1800947 (G > C), rs1130864 (C > T) and rs1205 (C > T).

Non-neuritic SP are diffuse and primitive SP grouped together, neuritic SP are classic and burnt out SP grouped together; as measured by a neuropathologist.

Prev % refers to prevalence of alleles.

Assoc. refers to associations with CRP levels.

CRP = c-reactive protein gene, SP = senile plaques, N = Number of cases, OR = odds ratio, CI = confidence interval, *p *= p value.

Haplotype pair analyses compared all haplotype pairs with prevalence above 6% against the most common pair (TTGTC/TCGCT). None of the haplotype pairs were associated with SP prevalence. Analyses with SP phenotype suggested a trend towards protection for the haplotype pair TTGTC/TTGTC (p = 0.065) and TCGCT/CCGCC (p = 0.070) with non-neuritic SP, although the association weakened with the inclusion of age and *APOE4 *carriership as covariates (data not shown).

NFT prevalence (yes/no presence) showed an association only with SNP rs2794521, using univariate logistic regression (p = 0.059). Inclusion of *APOE *genotype and age as covariates weakened the association (p = 0.107).

Semi-quantitative analyses of SP density did not reveal any significant associations with any of the *CRP *genotypes, and splitting the data by gender did not provide any additional results (data not shown).

### Immunohistochemistry

**Figure 1:**
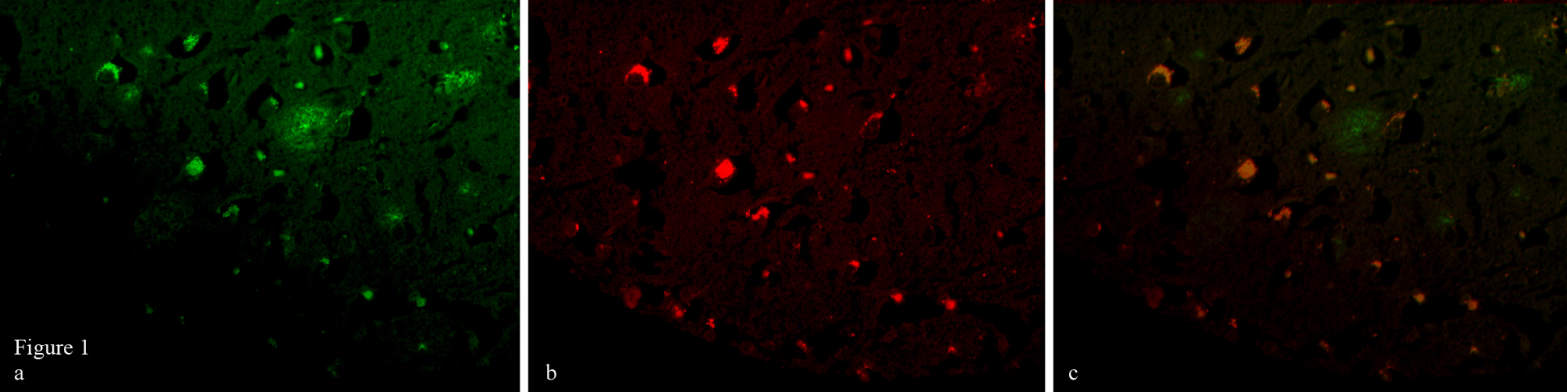
**Co-localisation of CRP and Aβ immunohistochemical staining (a) Aβ staining (b) CRP staining (c) merge, 100 × magnification**.

**Figure 2:**
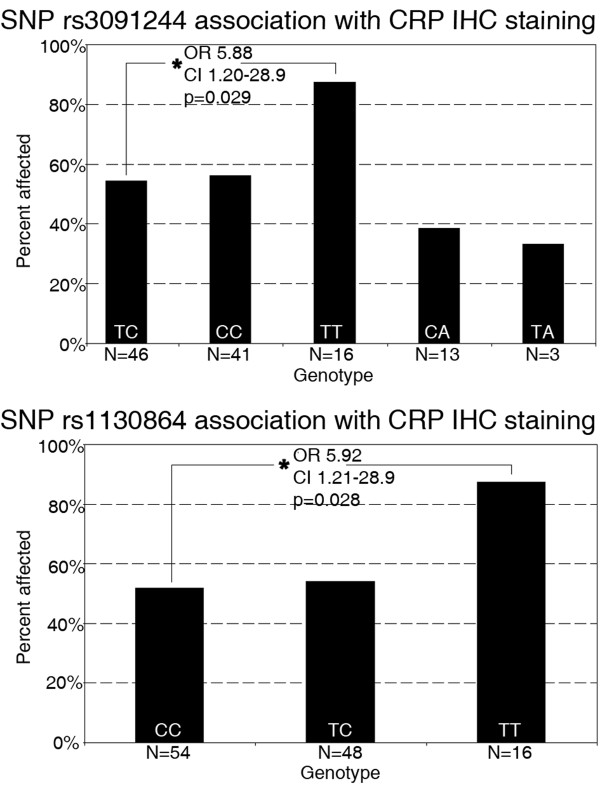
***CRP *SNPs and prevalence of CRP immunohistochemical staining (positive/negative) with SNPs rs3091244 and rs1130864**. Genotypes in order of population frequency, with * referring to 'no CRP staining' versus 'positive staining' with most common genotype as reference group.

CRP IHC staining (positive/negative) was found to be significantly correlated with Aβ (amyloid-β) staining (positive/negative) in all studied brain regions in the cohort, (Chi square p < 0.0001, Figure 1). Aβ IHC staining, however, was not found to be associated with any of the CRP SNPs or haplotypes. In univariate analyses, CRP IHC staining was significantly associated with high-CRP level TT genotypes of SNPs rs3091244 (OR 5.9, CI 1.20 - 28.87, p = 0.029) and rs1130864 (OR 5.9, CI 1.21 - 28.95, p = 0.028) (Figure 2). Individual haplotype (yes/no carriership) were not, but the haplotype pair TTGTC/TTGTC was significantly associated (OR = 5.5, CI = 1.03 - 29.48, p = 0.047) with CRP IHC staining. This relationship strengthened on inclusion of *APOE4 *carriership and age as covariates (OR = 14.9, CI = 1.14 - 196.37, p = 0.040), however the CI were extremely large.

### Multiple testing correction

We performed FDR calculations on our results, assuming that 11 independent tests were performed (6 SNPs and 5 haplotypes). These showed that with an FDR < 0.05, or 5% false positives, most of our results were still applicable (see Table 4). The SNPs and haplotypes of the *CRP *gene which were seen most often in analyses were rs2794521 (genotype CT), rs3091244 (genotypes TA and TT), rs3093075 (genotype CA) and haplotype TAGCC.

**Table 4:** Results validated by FDR < 0.05 cutoff limit

p-value	SNP (and genotype) or Haplotype	Association
p< 0.0001	n/a	Aβ IHC and CRP IHC stainings (Chi square)
p = 0.003	rs3093075 (genotype CA)	Increased risk of non-neuritic SP
p = 0.007	rs3091244 (TA)	Increased risk of non-neuritic SP
p = 0.007	Haplotype (6) TAGCC	Increased risk of non-neuritic SP
p = 0.037	rs2794521 (CT)	Reduced risk of non-neuritic SP
p = 0.076	rs1130864 (TT)	Reduced risk of non-neuritic SP
p = 0.076	Haplotype (4) TCGCT	Reduced risk of having NFT
p = 0.080	rs3093075 (CA)	Increased risk of neuritic SP
p = 0.083	rs2794521 (CT)	More likely to have CRP IHC staining
p = 0.087	rs3093075 (CA)	Less likely to have CRP IHC staining
p = 0.090	Haplotype (6) TAGCC	Less likely to have CRP IHC staining
p = 0.112	rs3091244 (TT)	Reduced risk of non-neuritic SP
p = 0.118	rs2794521 (CT)	Reduced risk of neuritic SP

## Discussion

The mechanisms underlying AD have been sought for more than 100 years, with not more than a few risk factors being identified, and the development of therapeutics has been based on treating symptoms, rather than reversing or curing the disease. Increasing population and average lifespan will see the number of AD sufferers escalate, according to current estimates, which will stress healthcare and treatment services.

Common understanding relates SP (aggregations of amyloid-β (Aβ) protein) and NFT (accumulations of hyperphosphorylated tau protein) in the brains of AD subjects as causes of the disease, with both triggering inflammation and disrupting neuronal signalling, and SP also implicated in genetic mutations of familial AD [3]. Our recently published study [28] on the prevalence of these brain lesions suggests that they are much more frequent, and occur in younger individuals, than previously thought, although whether the disease process also begins earlier is yet to be ascertained.

The inflammation theory was developed after epidemiological studies revealed a 6-times smaller incidence of AD in a cohort of patients receiving NSAIDs for rheumatoid arthritis, compared to a control group [10,33]. Whilst the effectiveness of NSAIDs is controversial in the treatment of AD [33], there is still a common consensus that inflammation is an important part of the AD process.

CRP is an acute phase inflammatory marker found in plasma. CRP levels have been shown to be upregulated in affected areas of AD brains [20]. Polymorphisms in the *CRP *gene associated with elevated CRP levels have been shown to increase mortality [22]. Research has implicated genetic factors as determining 27-40% of variance in plasma CRP levels [24,25].

A relationship between *CRP *genotype and NFT was not seen in our cohort, as was also the case in our earlier study of *APOE *genotype [28]. NFT formation is presumed to be secondary to SP production [34]; thus the lack of an association with *CRP *genotypes and NFT and the idea that *CRP *polymorphisms would be related only to SP is consistent.

The findings of our current work that some high-CRP level polymorphisms correlate with early non-neuritic SP allows us to hypothesise that increased inflammatory levels may initiate or participate in the primary development of lesions, which then leads to other processes and damage to neurons, thus setting off a chain of events leading to AD. The absence of statistically significant associations between *CRP *genotypes and late-stage neuritic SP could be due to other factors acting upon SP development, such as effects of immune cells, including microglia [35,36].

SNP rs2794521 has been previously reported to affect expression levels of CRP, with the T allele increasing transcription levels of the protein [24,25] compared to the C allele. In our cohort, this was the only SNP that associated with the occurrence of SP, with the most common CT genotype showing borderline significance for an association with reduced risk of having at least one SP (p = 0.067). When we further analysed the associations, taking into account early or late SP phenotype, we found that *CRP *SNP rs2794521 (C carriers) was significantly associated with reduced risk of harbouring non-neuritic SP. It may be possible that the CT genotype associates with lower levels of CRP, thus interfering with formation of SP. In contrast, high-CRP level SNPs (rs3091244, TA carriers and rs3093075 CA carriers) were strongly associated with increased risk of non-neuritic SP. However as a sign of the complex relationship between SNPs and CRP levels, we found that other high-CRP level SNPs, rs1130864 (TT carriers) and rs1205 (CC carriers), also showed trends toward protection against non-neuritic SP compared to no SP. These results nonetheless suggest a role for the *CRP *gene, independent of *APOE *genotype, which was used as a covariate in these analyses.

The CCGCC haplotype contains the protective, low-CRP protein-linked C allele for both rs2794521 and rs3091244, whilst TAGCC has the high-CRP level T and A alleles for the same SNPs. The effects of these SNPs were corroborated in haplotype analyses showing that CCGCC carriership reduces risk and TAGCC carriership increases risk for non-neuritic SP, with tendencies in the same directions for neuritic SP compared to no SP. Our results, showing a correlation between CRP and Aβ IHC staining, support the involvement of inflammation in AD and correspond with other studies [20].

In line with previous reports and with our results above, the high-CRP SNP rs3091244 (TT genotype) was significantly associated with CRP IHC staining in the CA1/2 region. In contrast, the previously reported high-CRP level TT genotype of rs1130864 was significantly associated with positive staining, although our SP results would suggest it has some protective effect in non-neuritic SP formation. This could suggest that this SNP may confer more effective clean-up abilities, and that higher levels, in this case, are not detrimental.

The absence of an association between Aβ staining and *CRP *genotype could be explained if CRP affects only SP formation and not the presence of the Aβ peptide itself, which is the product of normal amyloid precursor protein processing [37]. This makes sense, given the revealed associations between *CRP *genotypes and SP types in our study.

As the majority of the TASTY series are non-AD cases, correlative findings between *CRP *genotypes and SP prevalence reveal an interesting insight into the early development of AD neuropathology. It is possible that these SP-positive cases could be in a prodromal phase of the disease and may later have developed AD, had they lived. We recently showed, however, that 31% of the subjects in this series harbour SP, and that this prevalence increased to almost 100% in the oldest old. This questions the relevance of SP prevalence and the relationship between these brain lesions and AD itself.

Our data suggest that *CRP *genotype may modify initial SP formation in the brain. This is an interesting finding that will need to be investigated further in cohorts comprising only of AD cases, and replicated in larger epidemiological studies. It may be that *CRP *polymorphisms associate with or participate in the slowing down or enhancement of early stage SP but, after this, other factors come into play to effect conversion to late-stage SP. As end-stage SP are more likely to be associated with dementia than other types [34], this could explain why NSAID treatments in clinical AD patients have proven ineffective at slowing or reversing the disease, as inflammation may already have played its part. Based on our studies and others' results, the brains of most middle-aged to elderly persons possess some degree of persistent inflammation as well as SP and NFT. It could therefore be assumed that other factors aside from *CRP *genotype participate in the conversion of these 'benign' SP, to pathological SP types related to AD.

Whilst it may be that the younger aged cases and consequential low numbers of SP may reduce power, and may have caused some of our results to represent false positives, our cohort is a large autopsy series, showing the prevalence of these brain lesions in a sample representative of a general non-institutionalised population.

## Conclusions

The common occurrence of these AD-related brain lesions and the subclinical elevations in elderly patients of inflammatory markers [38], as well as our current results, suggest that these are simply a consequence of brain aging without any relationship to clinical AD. The conversion of these pathways into those causing AD, however, are yet to be ascertained and remain controversial.

## Abbreviations

AD: Alzheimer's disease; APOE: apolipoprotein E; CRP: C-reactive protein; FDR: false discovery rate; NFT: neurofibrillary tangles; NSAIDs: non-steroidal anti-inflammatory drugs; SNPs: single nucleotide polymorphisms; SP: senile plaques; TASTY: Tampere autopsy study; TMAs: tissue microarrays.

## Competing interests

The authors declare that they have no competing interests.

## Authors' contributions

All authors contributed to this manuscript. EK performed experiments and analyses and wrote the manuscript. MAK participated in writing the manuscript and provided comments and discussions. KI performed experiments. HH, TL and SH measured the neuropathological lesions. SG and PJK collected the autopsy series. MP, MH, HH and PJK provided comments and discussions on the progress of the manuscript. All authors have read and approved the final version.

## References

[B1] Pei J Sjogren M Winblad B Neurofibrillary degeneration in Alzheimer's disease: from molecular mechanisms to identification of drug targets Curr Opin Psychiatry 2008 21 555 561 10.1097/YCO.0b013e328314b78b 18852562

[B2] Kim Y Lim S Rhee S Park K Kim C Choi B Resveratrol inhibits inducible nitric oxide synthase and cyclooxygenase-2 expression in beta-amyloid-treated C6 glioma cells Int J Mol Med 2006 17 1069 75 16685418

[B3] Tanzi R Kovacs D Kim T Moir K Guenette S Wasco W The gene defects responsible for familial Alzheimer's disease Neurobiol Dis 1996 3 159 168 10.1006/nbdi.1996.0016 8980016

[B4] Kidd PM Alzheimer's disease, mild cognitive impairment amnestic and age-associated memory impairment: current understanding and progress toward integrative prevention Altern Med Rev 2008 13 85 115 18590347

[B5] Corder E Saunders A Strittmatter W Schmechel D Gaskell P Small G Gene dose of apolipoprotein E type 4 allele and the risk of Alzheimer's disease in late onset families 1993 261 Science (Washington) 828 9 10.1126/science.8346443 8346443

[B6] van Duijn C Wehnert A Van Broeckhoven C Havekes LM de Knijff P Cruts M Apolipoprotein E4 allele in a population-based study of early-onset Alzheimer's disease Nat Genet 1994 7 74 8 10.1038/ng0594-74 8075646

[B7] Beecham GW Martin ER Li YJ Slifer MA Gilbert JR Haines JL Genome-wide Association Study Implicates a Chromosome 12 Risk Locus for Late-Onset Alzheimer Disease The American Journal of Human Genetics 2009 84 35 43 10.1016/j.ajhg.2008.12.008 PMC2668056 19118814

[B8] Harold D Abraham R Hollingworth P Sims R Gerrish A Hamshere ML Genome-wide association study identifies variants at CLU and PICALM associated with Alzheimer's disease Nat Genet 2009 41 1088 U61 10.1038/ng.440 19734902 PMC2845877

[B9] Lambert JC Heath S Even G Campion D Sleegers K Hiltunen M Genome-wide association study identifies variants at CLU and CR1 associated with Alzheimer's disease Nat Genet 2009 41 1094 U68 10.1038/ng.439 19734903

[B10] McGeer PL McGeer E Rogers J Sibley J Anti-inflammatory drugs and Alzheimer disease Lancet 1990 335 1037 1970087 10.1016/0140-6736(90)91101-f

[B11] Breitner JCS Haneuse SJPA Walker R Dublin S Crane PK Gray SL Risk of dementia and AD with prior exposure to NSAIDs in an elderly community-based cohort Neurology 2009 72 1899 10.1212/WNL.0b013e3181a18691 19386997 PMC2690966

[B12] Holmes C Cunningham C Zotova E Woolford J Dean C Kerr S Systemic inflammation and disease progression in Alzheimer disease Neurology 2009 73 768 774 10.1212/WNL.0b013e3181b6bb95 19738171 PMC2848584

[B13] Lee K Chung J Choi T Suh S Oh B Hong C Peripheral cytokines and chemokines in Alzheimer's disease Dement Geriatr Cogn Disord 2009 28 281 7 10.1159/000245156 19828948

[B14] Perry V Nicoll J Holmes C Microglia in neurodegenerative disease Nat Rev Neurol 2010 6 193 201 10.1038/nrneurol.2010.17 20234358

[B15] Itzhaki RF Wozniak MA Herpes simplex virus type 1, apolipoprotein E, and cholesterol: a dangerous liaison in Alzheimer's disease and other disorders Prog Lipid Res 2006 45 73 90 10.1016/j.plipres.2005.11.003 16406033

[B16] Urosevic N Martins R Infection and Alzheimer's disease: the APOE epsilon4 connection and lipid metabolism J Alzheimer's Dis 2008 13 421 35 18487850 10.3233/jad-2008-13407

[B17] Kamer A Craig R Dasanayake A Brys M Glodzik-Sobanska L de Leon M Inflammation and Alzheimer's disease: Possible role of periodontal diseases Alzheimers Dement 2008 4 242 250 10.1016/j.jalz.2007.08.004 18631974

[B18] Pepys M Hirschfield G C-reactive protein: a critical update J Clin Invest 2003 111 1805 12 12813013 10.1172/JCI18921 PMC161431

[B19] Garlanda C Bottazzi B Bastone A Mantovani A Pentraxins at the crossroads between innate immunity, inflammation, matrix deposition, and female fertility Annu Rev Immunol 2005 23 337 366 10.1146/annurev.immunol.23.021704.115756 15771574

[B20] Yasojima K Schwab C McGeer E McGeer P Human neurons generate C-reactive protein and amyloid P: upregulation in Alzheimer's disease Brain Res 2000 887 80 10.1016/S0006-8993(00)02970-X 11134592

[B21] Eklund C Kivimaki M Shaheenul Islam M Juonala M Kahonen M Marniemi J C-reactive protein genetics is associated with carotid artery compliance in men in The Cardiovascular Risk in Young Finns Study Atherosclerosis 2008 196 841 8 10.1016/j.atherosclerosis.2007.01.027 17350021

[B22] Hurme M Kivimaki M Pertovaara M Lehtimaki T Karhunen PJ Jylha M CRP gene is involved in the regulation of human longevity: A follow-up study in Finnish nonagenarians Mech Ageing Dev 2007 128 574 576 10.1016/j.mad.2007.07.004 17765290

[B23] Ridker PM Loci related to metabolic-syndrome pathways including LEPR, HNF1A, IL6R, and GCKR associate with plasma C-reactive protein: the Women's Genome Health Study Am J Hum Genet 2008 82 1185 10.1016/j.ajhg.2008.03.015 18439548 PMC2427311

[B24] Teng M Hsu L Wu S Change H Choi H Ko Y Association between C-reactive protein gene haplotypes and C-reactive protein levels in Taiwanese: interaction with obesity Atherosclerosis 2009 204 e64 9 10.1016/j.atherosclerosis.2008.10.034 19101671

[B25] Wang L Lu X Li Y Li H Chen S Gu D Functional analysis of the C-reactive protein (CRP) gene -717A > G polymorphism associated with coronary heart disease BMC Med Genet 2009 10 73 19624831 10.1186/1471-2350-10-73 PMC2723087

[B26] Brull D Serrano N Zito F Jones L Montgomery H Rumley A Human CRP gene polymorphism influences CRP levels: implications for the prediction and pathogenesis of coronary heart disease Arterioscler Thromb Vasc Biol 2003 23 2063 9 10.1161/01.ATV.0000084640.21712.9C 12842840

[B27] Lee C You N Song Y Hsu Y Manson J Nathan L Relation of genetic variation in the gene coding for C-reactive protein with its plasma protein concentrations: findings from the Women's Health Initiative Observational Cohort Clin Chem 2009 55 351 60 19095725 10.1373/clinchem.2008.117176 PMC2856608

[B28] Kok E Haikonen S Luoto T Huhtala H Goebeler S Haapasalo H Apolipoprotein E-dependent accumulation of Alzheimer disease-related lesions begins in middle age Ann Neurol 2009 65 650 7 10.1002/ana.21696 19557866

[B29] Ghebremedhin E Schultz C Braak E Braak H High Frequency of Apolipoprotein E ε4 Allele in Young Individuals with Very Mild Alzheimer's Disease-Related Neurofibrillary Changes Exp Neurol 1998 153 152 155 10.1006/exnr.1998.6860 9743577

[B30] Braak H Braak E Frequency of Stages of Alzheimer-Related Lesions in Different Age Categories Neurobiol Aging 1997 18 351 357 10.1016/S0197-4580(97)00056-0 9330961

[B31] Stephens M Smith NJ Donnelly P A New Statistical Method for Haplotype Reconstruction from Population Data Am J Hum Genet 2001 68 978 989 10.1086/319501 11254454 PMC1275651

[B32] Stephens M Donnelly P A comparison of bayesian methods for haplotype reconstruction from population genotype data Am J Hum Genet 2003 73 1162 9 10.1086/379378 14574645 PMC1180495

[B33] P Aisen s A Fitzpatrick L Ikram MA A DeStefano L Gudnason V Boada M The potential of anti-inflammatory drugs for the treatment of Alzheimer's disease Lancet Neurol 2002 1 279 84 10.1016/S1474-4422(02)00133-3 12849425

[B34] Duyckaerts C Delatour B Potier M Classification and basic pathology of Alzheimer disease Acta Neuropathol 2009 118 5 36 10.1007/s00401-009-0532-1 19381658

[B35] Fukumoto H Asami-Odaka A Suzuki N Iwatsubo T Association of A beta 40-positive senile plaques with microglial cells in the brains of patients with Alzheimer's disease and in non-demented individuals Neurodegeneration, Neurodegen 1996 5 13 7 10.1006/neur.1996.0002 8731377

[B36] Ohgami T Kitamoto T Shin R Kaneko Y Ogomori K Tateishi J Increased senile plaques without microglia in Alzheimer's disease Acta Neuropathol 1991 81 242 247 10.1007/BF00305864 2058362

[B37] Haass C Schlossmacher M Hung A Vigo-Pelfrey C Mellon A Ostaszewski B Amyloid beta-peptide is produced by cultured cells during normal metabolism Nature 1992 359 322 325 10.1038/359322a0 1383826

[B38] Gallicchio L Chang H Christo D Thuita L Huang H Strickland P Single nucleotide polymorphisms in inflammation-related genes and mortality in a community-based cohort in Washington County, Maryland Am J Epidemiol 2008 167 807 813 10.1093/aje/kwm378 18263601

